# Compass—Canada’s first child psychiatry access program: Implementation and lessons learned

**DOI:** 10.1371/journal.pone.0323199

**Published:** 2025-06-23

**Authors:** Vivian W. L. Tsang, Michelle C. Q. Lin, Natalie Huang, Sally Tsoi, Jack Hu, Sonja Sinclair, Priya Watson, Susan Baer, Roberto Sassi

**Affiliations:** 1 Department of Psychiatry, Faculty of Medicine, University of British Columbia, Vancouver, British Columbia, Canada; 2 Department of Chemical Biology, University of British Columbia, Vancouver, British Columbia, Canada; 3 Department of Integrated Sciences, University of British Columbia, Vancouver, British Columbia, Canada; 4 McMaster University, Ontario, Canada; 5 Compass Program, BC Children’s Hospital, Vancouver, British Columbia, Canada; 6 Division of Child and Adolescent Psychiatry, BC Children’s Hospital, Vancouver, British Columbia, Canada; Jawaharlal Institute of Postgraduate Medical Education and Research, INDIA

## Abstract

**Background:**

There is a lack of mental health and substance use providers for youth in BC, particularly in rural and remote areas. To address these gaps, Canada’s first child psychiatry access program, BC Children’s Hospital Compass Program, was developed in 2018 to support providers across the province in providing evidence-based mental health and substance use care to youth under 25. This article describes the program’s first five years and provides an overview of its creation, utilization, and clinical uses.

**Methods:**

Quantitative data collected by the Compass Program from September 2018 through September 2023 were analyzed. Participation and utilization of the service by providers in the province were analyzed and descriptive statistics, including means with standard deviations for quantitative variables have been used to describe demographic and other medical factors related to participants.

**Findings:**

A total of 2336 new providers have been enrolled since Compass’ inception. Number of clinical calls into Compass remained steady over the five-year period with an average of 1085 individual providers served per year. Service use is highest in Vancouver Coastal Region (27.3%), followed by Northern Health (21.4%), Interior (15.7%), Vancouver Island (14.5%), and Fraser (13.4%), and Yukon (0.3%). General practitioners make up over a third of all encounters (34.6%), followed closely by pediatrician encounters making up 27.5% of total encounters from 2018–2023. These two provider types comprise over 60% of all encounters over the 5-year timespan. Encounters with other provider types were less common, with the third most common encounter being Child and Youth Mental Health (CYMH) clinicians, totalling 8.6% of total encounters. 37.6% of encounters were for male patients and 42.9% for female patients with 6.8% reporting “Other” genders and 12.7% declining to answer. Medication concerns are the most common reason for accessing Compass, regardless of gender. Therapy questions, resource coordination issues, and diagnostic clarification followed in frequency, comprising a similar amount of consults.

Compass consultations have the potential to benefit three groups of people: the specific patient being consulted on, the provider requesting the consultation, as well as the provider’s colleagues who might benefit from peer consultation.

**Conclusions:**

Capacity building is important given Compass receives calls from rural and remote areas where there are no psychiatrists or child psychiatrists where general practitioners and clinicians regularly work with patients along the entire spectrum of mental health and substance use disorders.

## Introduction

Canadian provinces and territories are facing challenges related to a shortage of mental health specialists. The dearth of access to specialized child and adolescent psychiatrists and mental health providers is particularly striking in rural and remote communities, as most subspecialties are concentrated in urban settings. The province of British Columbia (BC) is divided into five geographical health authorities. These are Interior Health Authority (IHA), Fraser Health Authority, Vancouver Coastal Health Authority, Vancouver Island Health Authority (VIHA), and Northern Health Authority (NHA). There is also the First Nations Health Authority (FNHA) which is a provincial health authority. Areas located within FNHA, IHA, VIHA, and NHA all include rural and remote communities. Between 2011–2018, the prevalence of diagnosed mental health disorders and substance usage increased among Canadian youth and young adults [1]. These rates have been particularly alarming among young women; with the number of generalized anxiety diagnoses tripling (3.8% in 2012 compared to 11.9% in 2022) and the likelihood of having a major depressive episode doubling from 9.0% in 2012 to 18.4% in 2022.2 Despite the rapid increase in mental disorders among Canadian youth, access to mental health services has not been increasing at an adequate rate, leaving 36.6% of Canadians with mood, anxiety, or substance use disorders to have unmet healthcare needs [2].

The recommended ratio of child and adolescent psychiatrists per youth is 1:3,800 [3]. Currently, in British Columbia (B.C.), this ratio is approximately 1:12,000; it is estimated that only 15% of the 180,000 children in BC who are in need of mental health services annually are able to access specialized care [3]. As a result, the majority of youth in the province receive their mental health care from their family physician, most of whom do not feel adequately prepared to take on the role of primary mental health provider [4]. Furthermore, mental health counsellors and psychologists are also primarily concentrated in urban areas [5]. Consequently, waitlists can be particularly long in rural and remote areas, and mental health can intensify in acuity. Therapists are often charged with treating a vast array of presenting issues, some of which may not align with their specialty areas.

The increased need for community support in addressing child and youth mental health and substance use care is also evidenced by the documented increases in emergency room presentations for mental health concerns in youth across North America from 3.1% to 8.6% annually [6]. A novel solution was needed in order to address the growing and complex needs of youth in B.C. struggling with mental health and substance use concerns, and support family physicians as well as other community-based clinicians in delivering evidence-based specialized mental health and substance use care.

Other countries have faced similar disparities and inequities related to mental health care [7,8]. Close and timely collaboration between primary care and mental health specialists has demonstrated effectiveness [9,10]. One of the first Child Psychiatry Access Programs (CPAP), aimed at facilitating these primary care-mental health specialist collaborations, was developed in Massachusetts in 2005 and there are now over 30 such programs in the United States [11,12]. These programs work to provide youth healthcare workers who care for individuals exhibiting mental health or behavioural symptoms with the necessary resources and information to best support their patients [13]. The Massachusetts Child Psychiatry Access Program (MCPAP) has proven successful in this method as it had an increase in utilization of 27% in 2021 compared to 2019, just two years prior [14]. This was in part thanks to the introduction of virtual health and telehealth visits due to COVID-19. In their 2021/2022 report, MCPAP utilization in 2021 was up 27% compared to pre-pandemic data from 2019 [14].

### Compass operations

#### Compass services.

Data identify key lessons learned in the development, implementation, and practice of this complex and busy CPAP.

This paper aims to provide an overview of BC Children’s Hospital’s Compass Program, Canada’s first CPAP. Compass is an interdisciplinary mental health consultation service that serves B.C. and the Yukon. Compass offers clinical support and consultation to community providers, including primary care physicians, pediatricians, and mental health clinicians. Regardless of diagnoses or clinical acuity, providers can seek one-time or ongoing clinical support and consultation about any patient up to age 25 presenting with mental health and/or substance use concerns. Compass also focuses on education, capacity building, and provider engagement activities, including webinar training, in-person outreach training programs, case-based learning series, and online toolkits and resources to support providers in assessing and treating common mental health concerns.

#### Staffing.

Compass operates as a transdisciplinary team and includes psychiatrists (2 FTE), psychiatric nurse clinicians (3.2 FTE), social workers (5 FTE), registered clinical counsellors (6 FTE), Indigenous care coordinator (1 FTE), Indigenous social workers (1 FTE), and a psychologist (0.8 FTE). The program is funded through the B.C. Ministry of Health and the Provincial Health Service Authority (PHSA), with occasional supplemental funding from private donors through BC Children’s Hospital Foundation, for online educational activities and outreach capacity-building initiatives. The consultation line is available 9:00 am to 5:00 pm, Monday through Friday. Seasoned clinicians answer the phone lines in real-time, with the occasional use of voicemail as needed. Follow-up phone or video consultations are then scheduled, as needed, with specific team members based on their particular areas of expertise.

## Methods

### Data collection

Data sources include quantitative data from the Compass RedCap database regarding patient demographics and presenting concerns, provider demographics, consultation questions, and data related to treatment recommendations and follow-up care.

The data presented in this study were collected on the Compass Redcap platform over a five-year period, beginning on the launch date of the Compass program (September 1^st^, 2018) and ending five years since then (August 31^st^, 2023). The data were grouped by year-long periods beginning in September and ending in August and represent the number of complete years of the program’s operation. The data were also further grouped by four 3-month quarters each year, beginning September 1st.

An encounter denotes a case concerning a patient with a particular issue and is distinguished from a call. An encounter can include an initial call and any number of follow-up calls regarding the same case. A particular patient may have more than one encounter recorded in the database.

Encounters are geographically associated with one of the five BC regional health authorities (Fraser Health, Interior Health, Northern Health, Vancouver Coastal Health, and Vancouver Island Health Authority) or Yukon. Population data were obtained from Statistics Canada’s 2016 Census of Population. Encounters or calls where the variables of interest were missing were excluded from the corresponding visualizations.

### Data analysis plan

Data analysis was performed using R (Version 4.2.1), with visualizations created using ggplot2 (Version 3.4.2) for graphical representation. Descriptive statistics, including means, medians, standard deviations, confidence intervals, and proportions, were calculated to summarize demographic (e.g., patient age and gender), geographic (e.g., regional distribution), and consultation-related variables (e.g., reasons for accessing the Compass program and encounter complexity). Patient data were stratified into analytical categories, including age groups (1–6 years, 7–12 years, 13–18 years, and 19–24 years), by dividing the total age range (1–24 years) into four equal numerical intervals to ensure consistent statistical comparisons across the full age range served by Compass. While this approach provides a structured framework for analysis, we acknowledge that neurodevelopmental progression does not align with the chosen age intervals or occur in strictly equal numerical increments. Additional stratifications included gender (male, female, and other), reasons for consultation (e.g., medication concerns, resource coordination, diagnostic clarification), and Indigenous status, as self-reported. Subcategories with limited sample sizes, such as genders not listed as male or female, were collapsed into an “other” category to ensure sufficient statistical representation.

To assess regional variations in utilization, encounter data were normalized per 1,000 inhabitants aged 0–24 years using the 2016 Census of Population for regional population estimates. Provider enrollment and encounter trends were examined over time using line graphs, where the x-axis represented time points (quarters or years) and the y-axis displayed the number of enrollments or encounters. The age distribution of patients was illustrated using a histogram to depict the frequency of encounters by age. Presenting concerns and reasons for accessing Compass were visualized using composite bar graphs and pie charts, with counts and proportions displayed for each category. Temporal trends in encounter volume and provider engagement were analyzed descriptively across quarterly intervals to highlight changes over the program’s five-year period. Encounter complexity was categorized as “single” (one documented reason for consultation) or “multiple” (two or more reasons). Proportions of single and multiple presenting complaints were calculated and compared across regions and provider types.

## Results

### Service utilization

**Fig 1:**
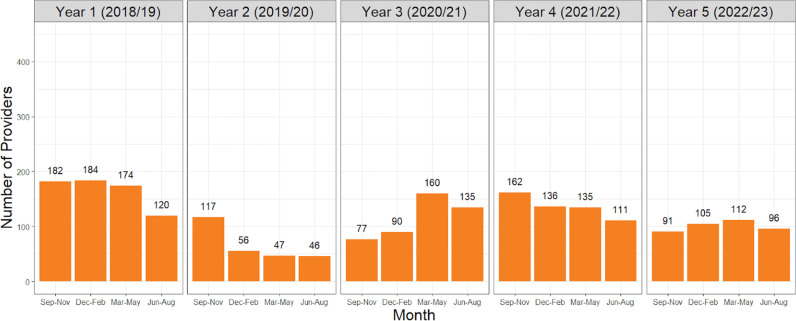
Number of new providers enrolled into the Compass program by quarter.

**Fig 2:**
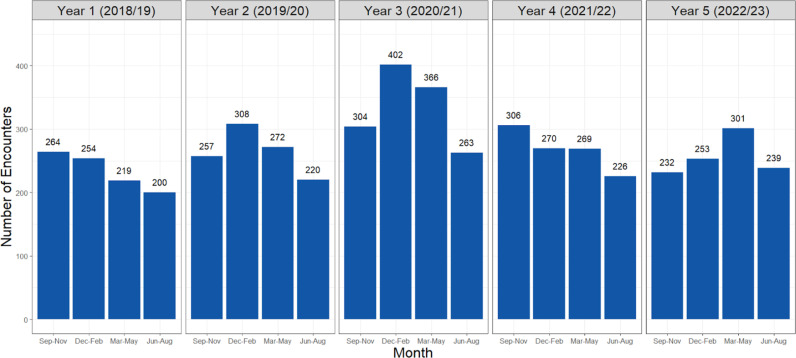
Total number of encounters per quarter.

The Compass program has enrolled over 2,336 providers and managed more than 5,709 cases during its first five years of operation beginning in September 2018. Program adoption and utilization were assessed through quarterly provider enrollment trends (Fig 1). The enrollment of new providers into the program spiked at launch, declined in Year 2, and stabilized between Years 4 and 5, with a total of 2,336 providers enrolled. Encounter numbers are relatively stable, averaging 1,085 encounters annually with a peak in winter of Year 3 (Fig 2).

**Fig 3:**
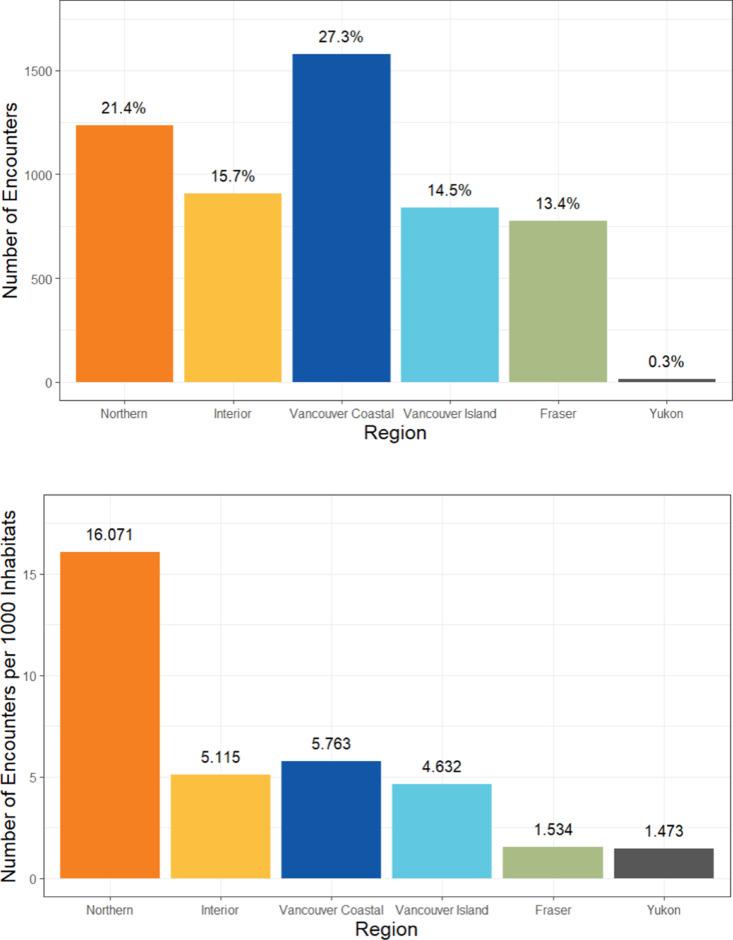
(A-B). A summary of the encounters per region served. (2018–2023).

Although Vancouver Coastal has the highest absolute number of total encounters, Northern regions show higher frequency when accounting for population differences (Fig 3).

**Fig 4:**
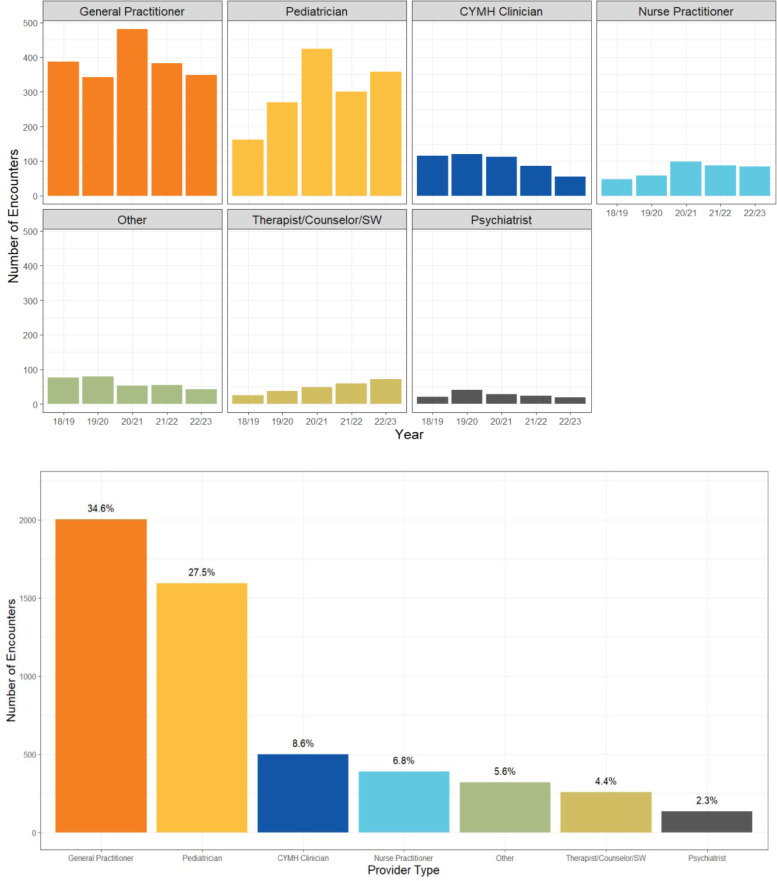
(A-B). A summary of the encounters by provider type. (A) Total number of encounters from 2018 to 2023. (B) Annual breakdowns.

General practitioners (34.6%) and paediatricians (27.5%) collectively constitute over 60% of encounters from 2018 to 2023. Encounters involving Child and Youth Mental Health (CYMH) clinicians account for 8.6% but have steadily declined. In contrast, paediatrician encounters have increased, peaking in 2020/2021, while those with therapists, counsellors, and social workers (SW) have shown a steady increase. Encounters with general practitioners remain steady (Fig 4). CYMH clinicians are the most frequent callers in the Northern, Interior, and Fraser regions, whereas general practitioners are the primary callers in Vancouver Coastal, Vancouver Island, and Yukon. The ‘Other’ and ‘N/A’ categories were excluded due to data volume (supplementary S1 Fig).

### Patient demographics

**Fig 5:**
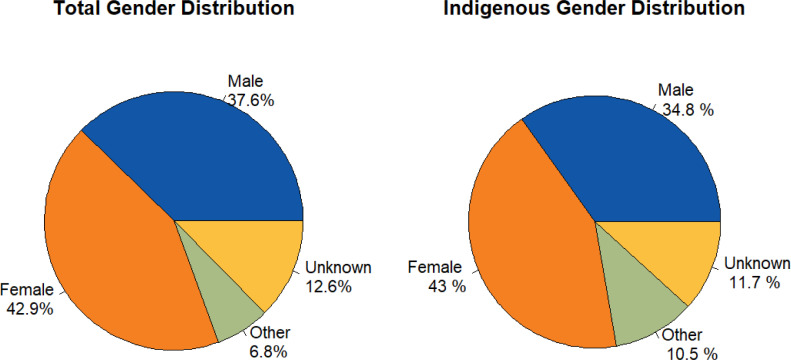
(A-B). Gender distribution of patients accessing Compass.

Female patients comprise 42.9% of Compass encounters. There is a similar gender distribution among indigenous patients (Fig 5).

The average age of patients utilizing the Compass program is 13.4 years old, with the highest number of encounters occurring with patients aged 15, followed by age 16 (supplementary S2 Fig). A notable decline in encounters is observed after age 17, which contributes to the overall mean age of 13.4 despite the highest number of encounters at age 15.

### Reasons for accessing care

**Fig 6:**
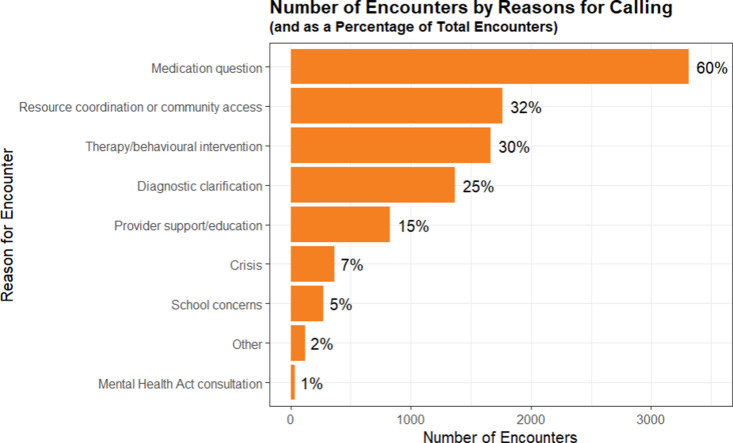
The number and percentage of encounters separated by reasons for calling.

Compass encounters most frequently occur for medication questions (60%), followed by 32% of encounters for resource coordination or community access and 30% of encounters for therapy/behavioural intervention (Fig 6). A quarter of encounters stated diagnostic clarification as their reason for calling, and 15% stated provider support/education as their reason for calling. Crises, school concerns, and consultation on the Mental Health Act make up 7%, 5%, and 1% of reasons for calling respectively, and 2% of encounters reported other reasons for calling.

Medication concerns are the most common reason for accessing Compass, regardless of gender, with a total of 2665 cases across all genders (1164 male encounters for medication questions; 1273 female encounters for medication questions; 228 reporting “other” genders for medication questions) (supporting information). Apart from medication questions, therapy questions (1309 total encounters; 531 male encounters; 607 female encounters; 171 encounters from patients reporting “other” genders), resource coordination (1293 total encounters; 515 male encounters; 622 female encounters; 156 encounters from patients reporting “other” genders), and diagnostic clarifications (1137 total encounters; 451 male encounters; 530 female encounters; 156 encounters from patients reporting “other” genders) have a similar amount of patients when comparing each gender. The number of males accessing Compass is higher than the number of females accessing Compass for school concerns (87 males; 79 females). This is different from the general trend where females make up the higher percentage of patients accessing Compass for most other reasons. Similarly, the number of patients reporting “Other” genders was also higher in terms of percentage when accessing Compass for school reasons than they were in other reasons for accessing Compass (24.2% of all encounters listing school concerns as their reason for accessing Compass were from patients listing “other” genders).

To determine the reasons for which patients accessed Compass in different age groups, data was separated into age groups 1–6, 7–12, 13–18, and 19–24 stratified by gender (supplementary S3 Fig). Among younger age groups (1–6 and 7–12 cohorts), there are more male patients than female patients in most categories, with male patients making up the majority in questions about medication, therapy questions, diagnostic clarifications, and school concerns in both categories. This pattern is reversed in the older age groups, where female patients accessing Compass are generally higher in number relative to male patients. In the older age groups, apart from resource coordination, patients identifying as female outnumber patients identifying as male. The primary reason for accessing Compass, regardless of age group, is for medication questions. However, secondary reasons for accessing Compass change depending on the age group subsequently. In the younger age groups (1–6 and 7–12), the second most common reason for accessing Compass is for therapy questions, while for older age groups (13–18 and 19–24), the second most common reason for accessing Compass is for resource coordination.

The majority of encounters have at least two presenting complaints, especially in rural and remote regions in BC. Vancouver Coastal Health region has the greatest number of single complaint encounters, which are most frequently medication concerns.

### Patient presenting concerns

**Fig 7:**
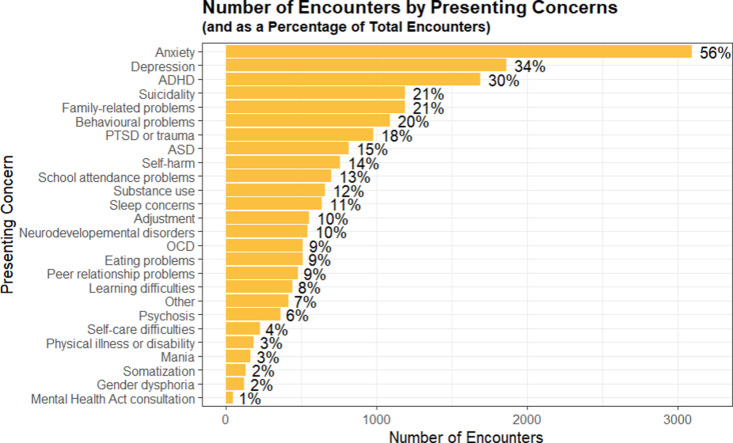
The number and percentage of encounters separated by the presenting concerns.

In terms of presenting concerns, the majority of encounters (56%) noted anxiety as the presenting concern, which can also be noted in the age group data (supplementary S4 Fig) show 34% of encounters reported depression as the presenting concern, 30% reported ADHD as the presenting concern, and 21% reported suicidality or family-related problems as the presenting concern. These numbers are supported by the age group data (Fig 7), where anxiety, depression, ADHD, suicidality, and family-related problems generally remain among the most common concerns seen in patients of all age groups.

ssupporting information shows the most common concern for males aged 1–6 is attention difficulties/hyperactivity, with 85 encounters. For females and those listing their gender as “other” in the same age group, the primary concern was disruptive/antisocial/aggressive behaviours (28 encounters for females, 13 encounters for “other”), followed by anxiety and attention difficulties/hyperactivity. In this age category (1–6), males have the highest number of concerns. When looking at the second age category (ages 7–12), the most common concern for males is attention difficulties/hyperactivity, with 381 encounters. For females and those identifying as “other”, the most common concern was anxiety (280 for females; 62 for “other”). Once again, males have the most number of concerns in this age category. In the third age group (patients aged 13–18), the most common concern for all genders is now anxiety (459 encounters for males, 913 for females, 149 for “other”). Females now have the highest number of concerns in this age categories, and among the top 10 most common concerns (anxiety, depression, suicidality, self-harm, family-related problems, attention difficulties/hyperactivity, PTSD, substance use concerns, eating problems, and sleep concerns), females make up the majority of the patients with these concerns, apart from attention difficulties/hyperactivity, where males continue to make up the majority. Finally, in the last age group (patients between 19–24 years old), the most common concern for all genders is again, anxiety (males with 33 encounters; females with 52; “other” people with 16). Females, once again, have the most number of concerns in this age category. However, among the top 10 most common categories, we see males making up a higher percentage in comparison to the 13–18 age group. Males make up the majority of concerns in substance use, attention difficulties/hyperactivity, ASD, and intellectual/neurodevelopmental disorders (supplementary S5 and S6 Figs).

## Discussion

Compass consultations have the potential to benefit three groups of people: the patient (i.e., subject of the consultation), the provider requesting the consultation, as well as the provider’s current/future patients who present with similar concerns and colleagues who might benefit from peer consultation. The mandate of Compass is designed to be three-pronged in consideration of these stakeholders [15]. In addition to providing psychiatric support for patients in BC who do not have direct access to a child psychiatrist, Compass also serves to build capacity among community providers. However, as shown from the regional distribution of providers (Fig 1), Compass routinely receives calls from family doctors or pediatricians in areas both with and without child psychiatrists. This speaks to the lack of capacity for child psychiatry services in British Columbia and within the rural areas of the Greater Vancouver region [16]. Mental health clinicians in remote areas are scarce, and are often required to address a wide spectrum of mental health and substance use disorders [5]. Despite approximately 84,000 children and youth aged 4–17 in British Columbia suffering from clinically significant mental disorders at any moment, there is a concerningly large discrepancy in the number of youth psychiatrists in BC [17,18]. This is evidenced by the current ratio of youth psychiatrists per youth in BC being 1:12,000 while the recommended ratio per CPA is 1:3,800 [19]. Low enrolment rates of providers in the second half of year 2 and the first half of year 3 directly correspond with the timing of COVID-19. A change in the delivery of mental health resources during this time was undertaken globally in an attempt to make mental health services more accessible. Face-to-face services decreased while the use of telemental health services increased to safely address mental health needs while following health guidelines [20]. Various studies have now captured the critical impacts of quarantine and isolation on the mental wellness of children and youth including depression, anxiety, and suicide among Canadian youth during this period [21]. There is a sharp increase in provider enrolment between March and May of 2021 again speaking to an increased need for child psychiatry resources as pandemic restrictions were removed (Fig 1). The upper threshold of around 160 provider enrollments per quarter post-pandemic speaks to the capacity of Compass as a program.

Post-pandemic, we also see a sharp rise in encounters per quarter with a peak of 402 encounters between December 2020 and February 2021 which again likely speaks to the mental health impacts of pandemic measures. Younger Canadian adults’ mental health were found to have been disproportionately affected by the COVID-19 pandemic compared to older age groups [22]. There were higher proportions of individuals below the age of 44 surveyed between fall of 2020 to spring 2021 that screened positive for depression and anxiety (from 18% to 23% and 15% to 20% respectively) [22]. Among Canadian individuals aged 18–24, a survey on mental health following the COVID-19 pandemic found that a significant proportion of respondents experienced moderate to severe symptoms of psychological disorders including depression (33%), anxiety (25%), and post-traumatic stress disorder (15%) [23]. When speaking about variations between different health authorities in BC, the number of absolute encounters being highest in Vancouver Coastal Health account for the largest absolute number of children and youth in the region. However, Fig 3B shows the stark need in Northern Health Regions with 16 encounters per 1000 inhabitants. This is nearly three times the proportion of encounters for other health authorities which speaks to the immense need and underserved population in this region.

### Capacity building

Capacity building is also the focus of outreach and training. It was anticipated that providing consultation and training for community providers would create an opportunity to build capacity. Results show that over 70% of providers that access the Compass program are prescribers. This includes general practitioners, paediatricians, and nurse practitioners. This may very well be because prescribers are able to bill for their consultation time with Compass and collect continuing education credits, which further supports prescribers taking time out of their already busy schedules to consult with Compass. This trend also correlates with the majority of encounters being for the purposes of medication review. Additionally, one of the main resources available at Compass is a provider to child psychiatrist direct consultation. Unique to the child psychiatrist role is expert knowledge on medications potentially explaining why over 60% of encounters cite this as at least one of their reasons for accessing Compass.

To further illustrate the importance of capacity-building initiatives, programs such as CanREACH, a licensee of the Resource for Advancing Children’s Health (REACH) Institute’s Pediatric Primary Care fellowship-training program, demonstrate how providing primary care providers with specialized, accessible training can enhance the delivery of child and adolescent mental health care [24]. CanREACH aims to increase participating care providers’ knowledge and skills in the identification, diagnosing, and treatment/monitoring of child and adolescents’ mental health [24]. The program emphasizes the development of self-efficacy and putting physicians’ newfound skills into clinical practice to ensure sustainability of learning post-program participation [24]. These initiatives align closely with the Compass program’s goal of capacity building and increasing provider confidence in delivering mental health care. Lessons from the CanREACH program may be incorporated into Compass’s strategies to further strengthen its outreach and enhance its impact, especially in underserved areas.

The distribution of provider types utilizing Compass varies by region. CYMH clinicians are the most frequent callers in the Northern, Interior, and Fraser regions, whereas general practitioners are the primary callers in Vancouver Coastal, Vancouver Island, and Yukon. This likely connects to the lack of physicians in rural and northern Canadian communities as over 20% of Canadians live in these communities despite only having access to 10% of physicians, 17% of family physicians, and 3% of specialists [25].

### Special populations

Compass generally serves more female patients. However, when evaluating the presenting concerns stratified by age, it is apparent that anxiety comprises the majority of concerns and that this is most prominent in females, especially in the 13–18 and 19–24 cohorts. The trends in populations and presenting concerns seen at Compass mirror those in outpatient child psychiatry services. In general, in the younger age cohorts of 1–6 and 7–12, presenting concerns are dominated by males for attention difficulties/hyperactivity and disruptive/antisocial/aggressive behaviours with these behaviours being young males being 2.3 times more frequent in comparison to young females [26]. Similarly, the presenting concerns of teenage females with anxiety also mirror those of outpatient psychiatric services. When examining differences in anxiety levels and symptoms of anxiety disorders between female and male students, it was found that female students scored higher anxiety levels in all three different major factors: physiological anxiety, worry and oversensitivity, and concentration. Over a total of students between the first to fifth grades, the mean score of female students was 18.87 compared to the mean male students’ score of 18.11 [27]

There are several learnings from the program to date. In particular, the reasons for accessing Compass support match the epidemiological patterns seen for psychiatric interventions at outpatient clinics at BC Children’s Hospital with medication questions topping the list (Fig 6). Moreover, the mean age of Compass encounters is 13.4 years, similar to the mean age of mental health-related visits at the BC Children’s Hospital Pediatric Emergency Department (PED) being 13.2 years between January 2002 and December 2012 [28].

Different from most other CPAPs, Compass is available to providers calling about children, adolescents, and young adults up to the age of 25 years old [15]. However, the number of consultations received pertaining to emerging adults is minimal in comparison to other age groups (Fig 6). There is a sharp decrease in encounters for young adults 18 and above. This is an important area of investigation and potential growth. This points to a possible consideration that community providers across the province may not recognize the wider span of support available through Compass for their adolescent and young adult patients. It is possible that general practitioners and other community providers working with young adults may not be aware of Compass, suggesting a need to alter marketing and communication strategies. It is also possible that many vulnerable young adults are lost to follow-up due to the relative lack of wrap-around and transitional services for this age group into adult care.

A mandate of Compass, unique from most other CPAPs, is to address both mental health issues and substance use concerns. By promoting integrated care, the goal is to increase access and remove barriers often experienced in siloed systems where mental health and substance use are addressed separately. However, compared to similar substance use services available in the province, Compass receives proportionally fewer primary or concurrent substance use calls. There may be a multifaceted rationale for this including a lack of knowledge from providers regarding the available service, comfort from primary health providers in addressing substance use, and alternative supports in addictions services in the community ranging from rehabilitation to outpatient programs.

When developing the Compass program, it was predicted that consultations would generally range from mild to moderate in severity. However, service utilization trends for Compass highlight that the majority of consultations received are often complex and comprise multiple domains of questions for Compass staff (Fig 11). Many complex cases also revolve around special population groups served by Compass. This has shed light on several gaps and limitations within the mental health care system.

### Limitations

Limitations to this publication include the necessary acknowledgement that all data presented are retrospectively analyzed. To this effect, the data collection process is part of clinical care. As such, there may often be missing fields due to clinicians not being able to access chart details, or discrepancies between clinician to clinician logging of each case. While training and standardized protocols mitigate these differences, this is an important limitation to the clarity and precision of data collection. In addition, Compass is a new and iterative program. Throughout its five years of existence, there have been many changes to the program through an iterative quality improvement approach. As such, it is difficult to completely standardize variables and confounders when analyzing and interpreting data. Furthermore, age stratification in this study was based on equal numerical intervals rather than neurodevelopmental stages, which may not fully capture differences in mental health presentation and service needs. Future studies may consider alternative age stratifications that align more closely with key neurodevelopmental milestones to provide a more developmentally informed analysis.

### Future directions

In the future, it will be important to compare Compass data and service outputs to similar CPAP programs across the world. In particular, it will be especially important to collaborate with MCPAP and other groups in the United States to compare trends in telepsychiatry visits in different regions of the world. In addition, specific prospective projects to collect and share evidence on community provider capacity building will be important in ensuring that Compass is meeting the goals underlying its creation.

## Supporting information

S1 Fig Frequency of consults by provider types by region. (DOCX)

S2 Fig The age distribution of Compass encounters. (DOCX)

S3 Fig Reasons for accessing Compass separated by gender. (DOCX)

S4 Fig (A-D). Reasons for accessing Compass separated by age and gender. Panel (A) shows reasons for accessing Compass for children aged 1–6, panel (B) shows reasons for accessing Compass for children aged 7–12, panel (C) shows reasons for accessing Compass for children aged 13–18, and panel (D) shows reasons for accessing Compass for people aged 19–24. Within each panel, encounters are separated by gender.
(DOCX)

S5 Fig Complexity of Compass encounters by region. (DOCX)

S6 Fig (A-D). Patient concerns separated by age range and gender. Panel (A) shows the most common patient concerns for patients aged 1–6, panel (B) shows the most common patient concerns for patients aged 7–12, panel (C) shows common patient concerns from those aged 13–18, and panel (D) shows the common patient concerns for those aged 19–24. Within each panel, concerns are separated by gender.
(DOCX)
