# Supplementary figures and images for: Compass—Canada’s first child psychiatry access program: Implementation and lessons learned
Source: PLoS One. 2025 Jun 23;20(6):e0323199. doi: 10.1371/journal.pone.0323199 (PMC12184907; doi:10.1371/journal.pone.0323199)

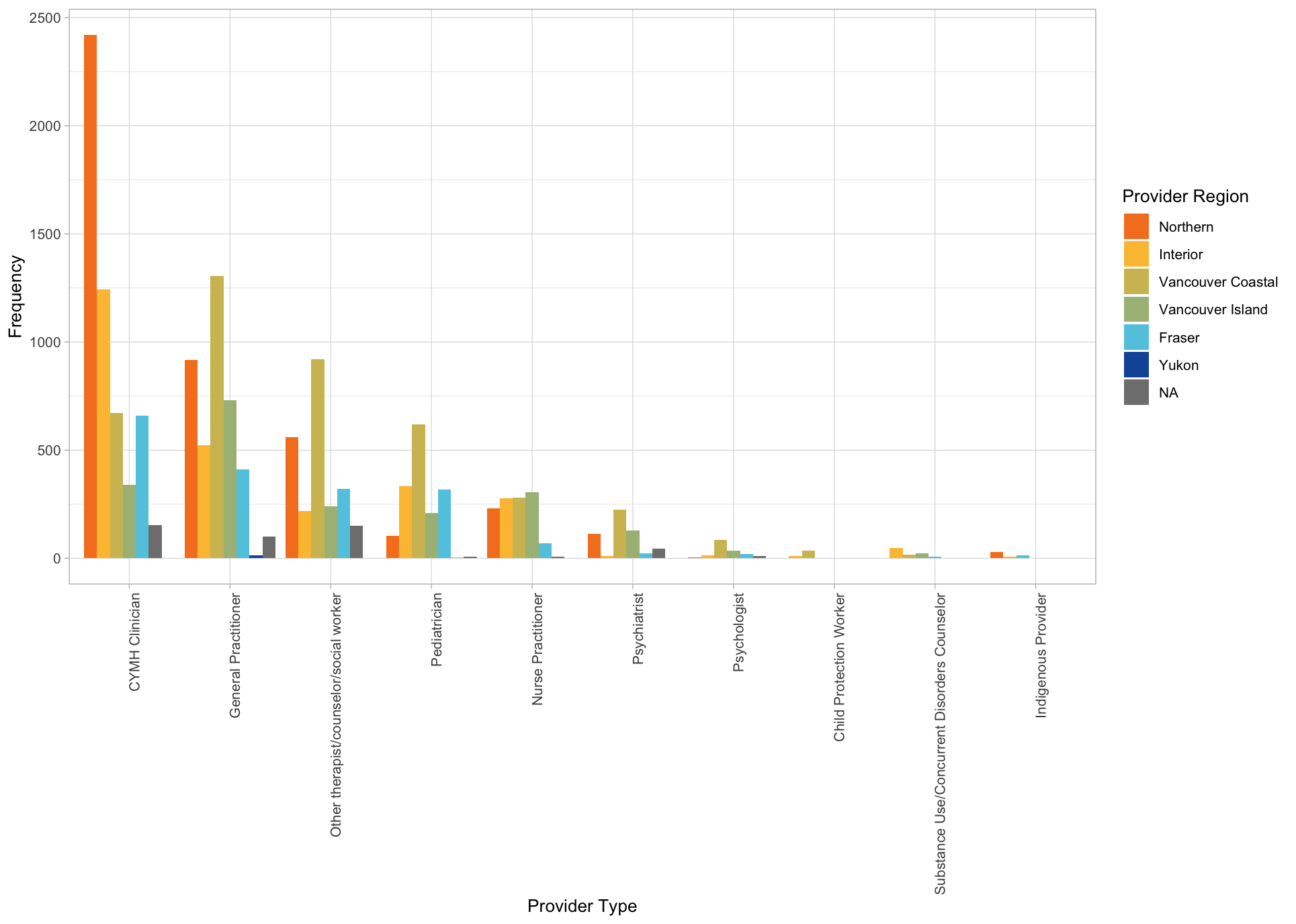


**Supplementary figure 1. Frequency of consults by provider types by region**

Supplement: S1 Fig — (DOCX) [file pone.0323199.s001.docx]

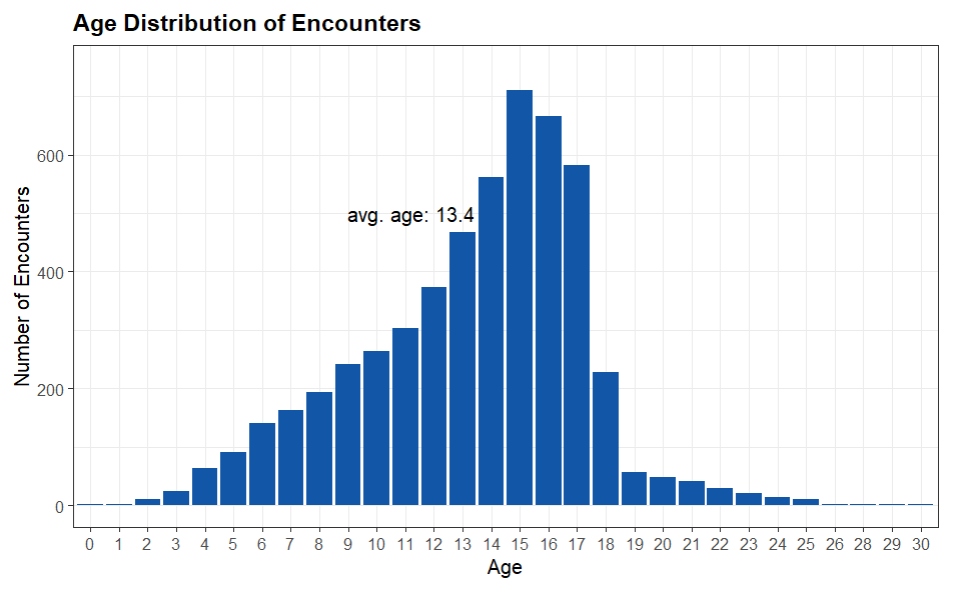


**Supplementary figure 2. The age distribution of Compass encounters**

Supplement: S2 Fig — (DOCX) [file pone.0323199.s002.docx]

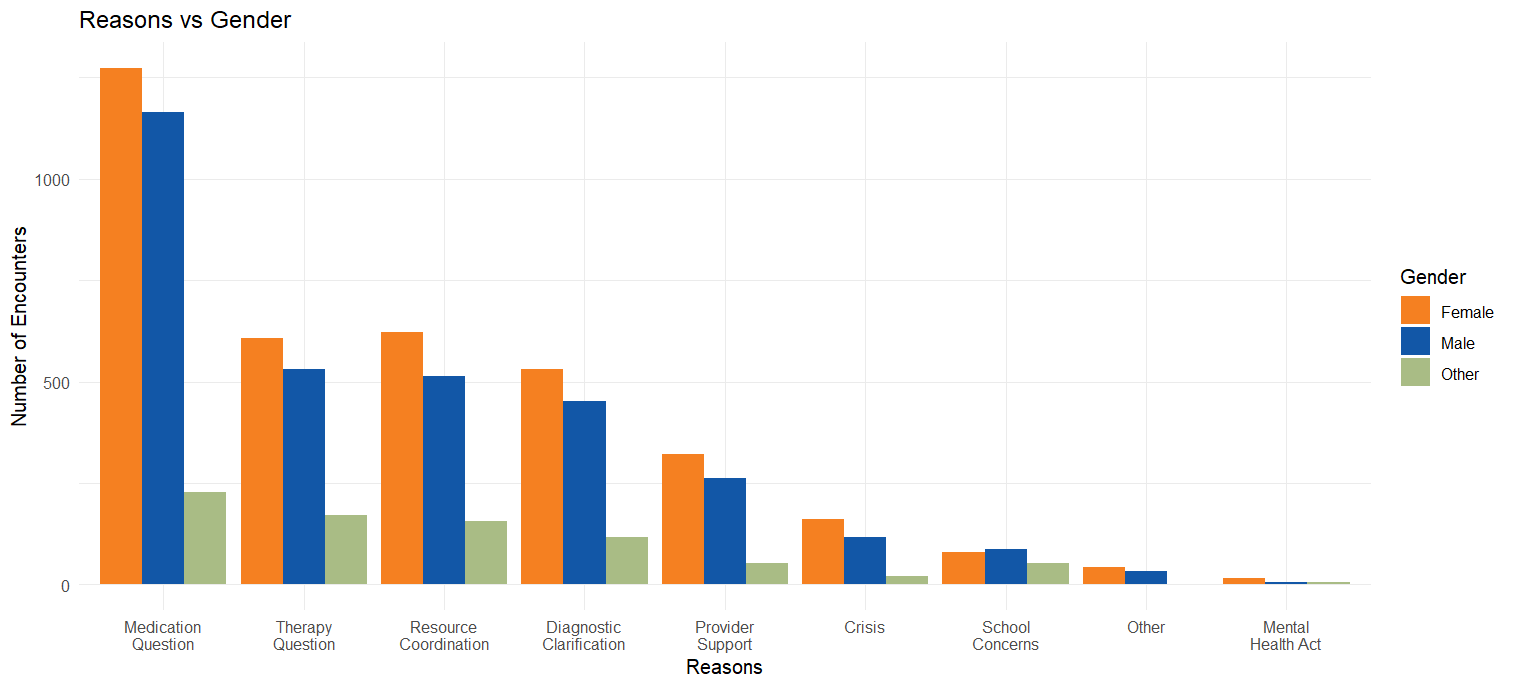


**Supplementary figure 3. Reasons for accessing Compass separated by gender**

Supplement: S3 Fig — (DOCX) [file pone.0323199.s003.docx]

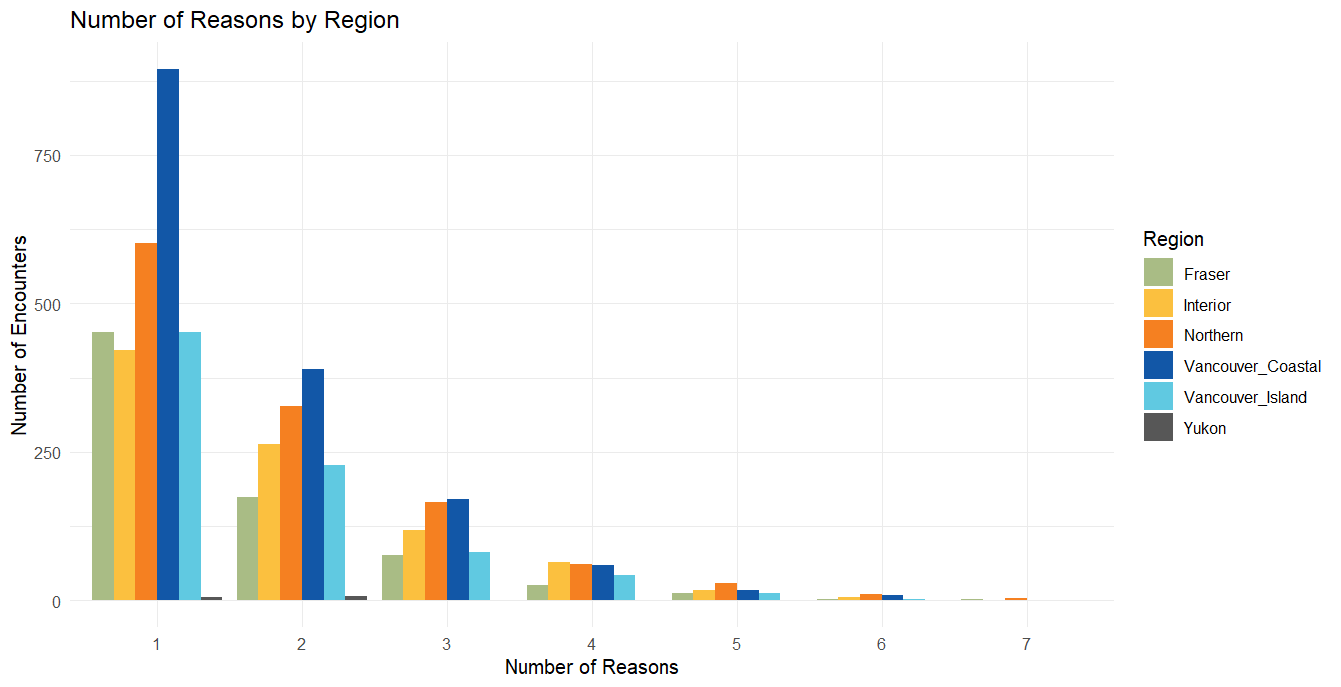


**Supplementary figure 5. Complexity of Compass encounters by region**

Supplement: S5 Fig — (DOCX) [file pone.0323199.s005.docx]
