# Supplementary material for: Compass—Canada’s first child psychiatry access program: Implementation and lessons learned
Source: PLoS One. 2025 Jun 23;20(6):e0323199. doi: 10.1371/journal.pone.0323199 (PMC12184907; doi:10.1371/journal.pone.0323199)
Supplement: S4 Fig — Panel (A) shows reasons for accessing Compass for children aged 1–6, panel (B) shows reasons for accessing Compass for children aged 7–12, panel (C) shows reasons for accessing Compass for children aged 13–18, and panel (D) shows reasons for accessing Compass for people aged 19–24. Within each panel, encounters are separated by gender. (DOCX) [file pone.0323199.s004.docx]

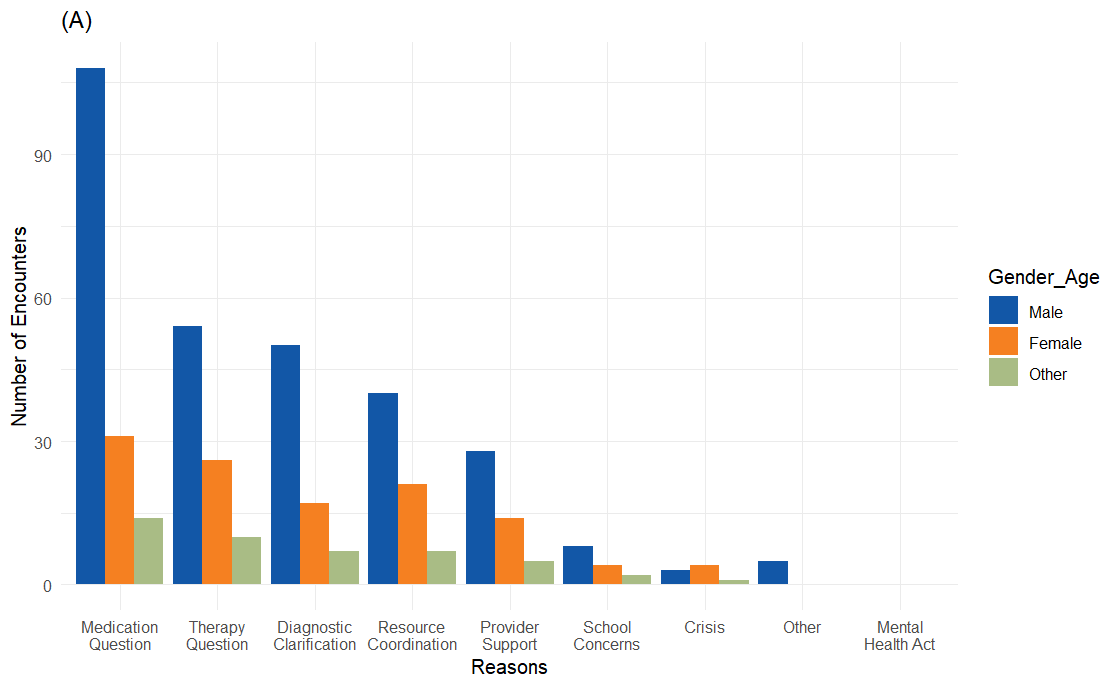

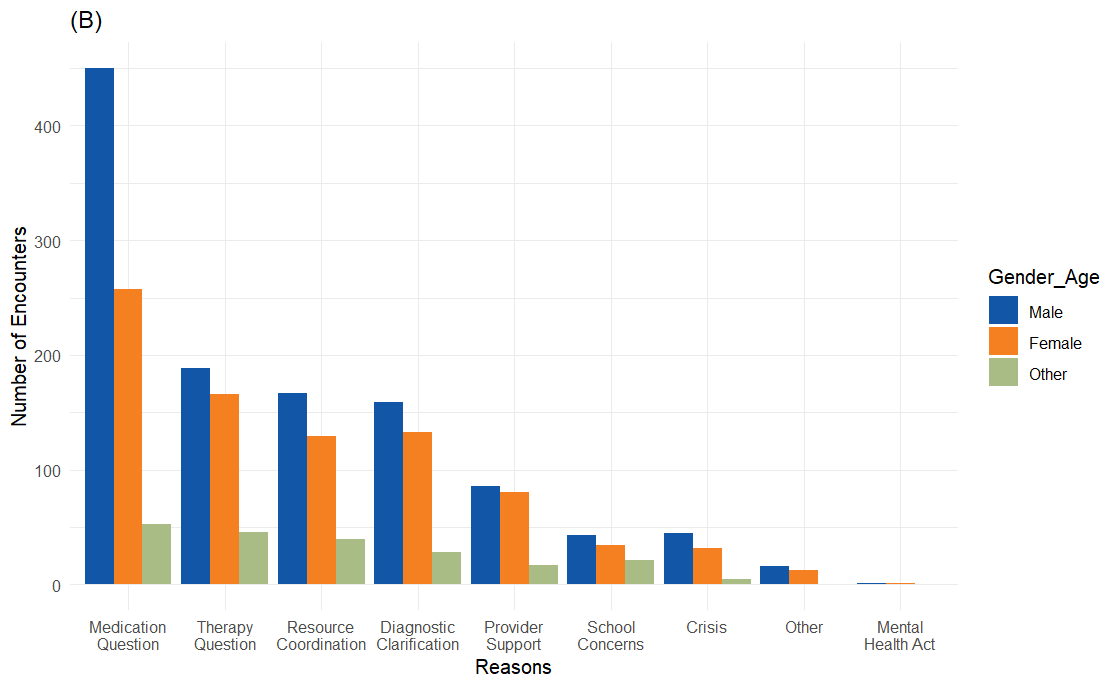

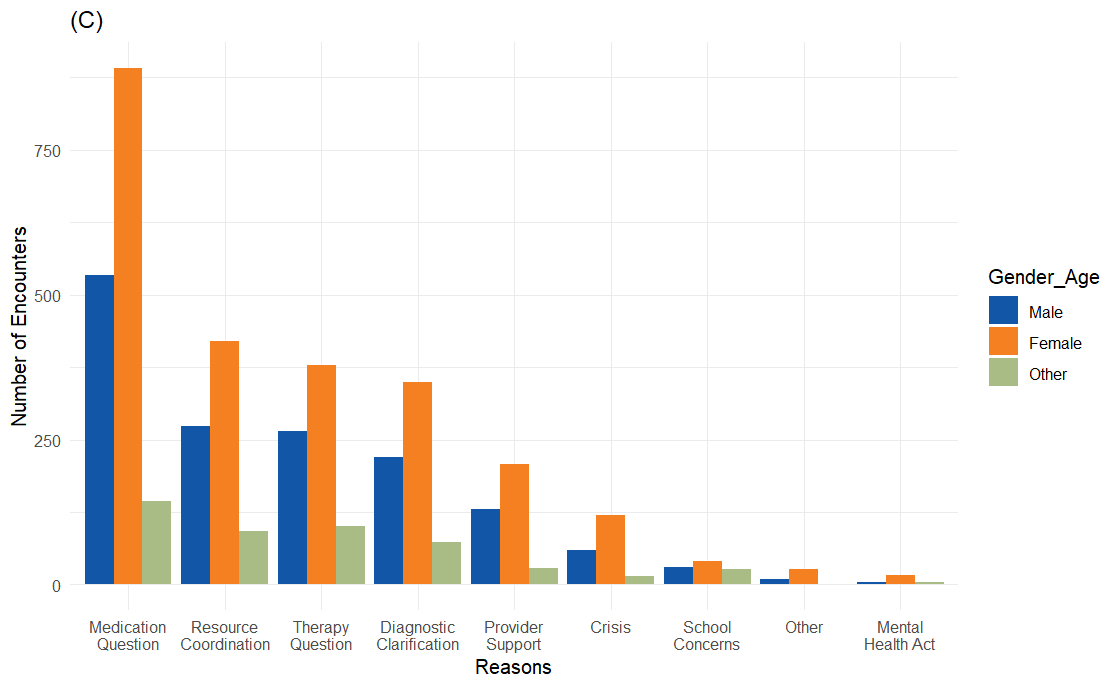

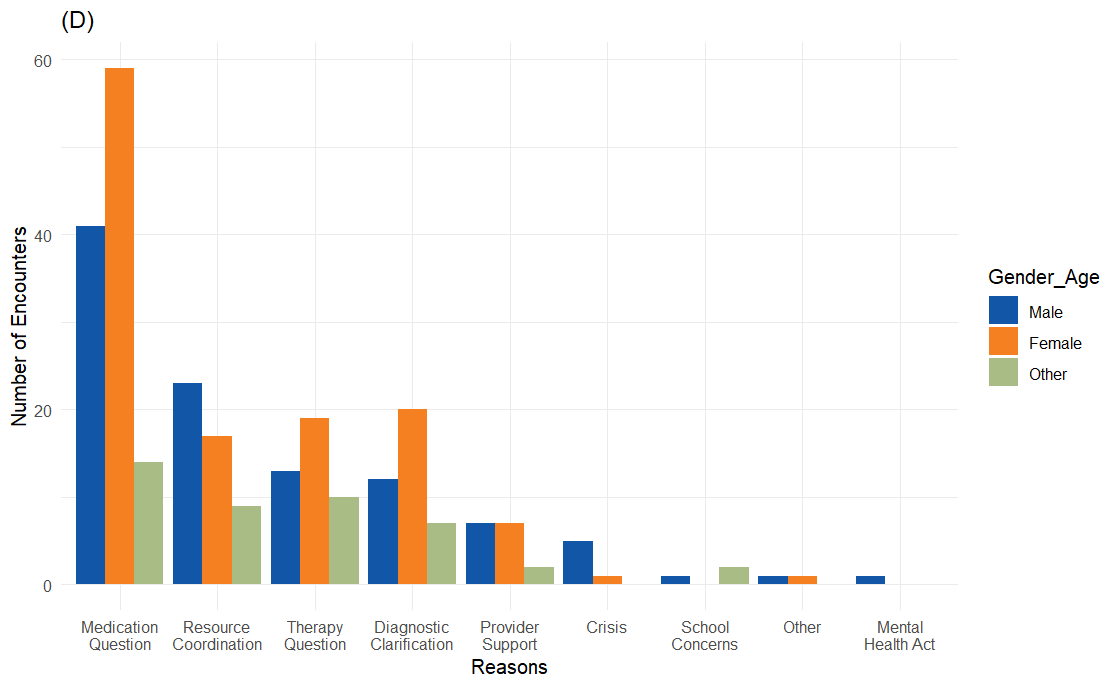


**Supplementary figure 4 (A-D). Reasons for accessing Compass separated by age and gender.** **Panel (A) shows reasons for accessing Compass for children aged 1-6, panel (B) shows reasons for accessing Compass for children aged 7-12, panel (C) shows reasons for accessing Compass for children aged 13-18, and panel (D) shows reasons for accessing Compass for people aged 19-24. Within each panel, encounters are separated by gender.**
