# Supplementary material for: Compass—Canada’s first child psychiatry access program: Implementation and lessons learned
Source: PLoS One. 2025 Jun 23;20(6):e0323199. doi: 10.1371/journal.pone.0323199 (PMC12184907; doi:10.1371/journal.pone.0323199)
Supplement: S6 Fig — Panel (A) shows the most common patient concerns for patients aged 1–6, panel (B) shows the most common patient concerns for patients aged 7–12, panel (C) shows common patient concerns from those aged 13–18, and panel (D) shows the common patient concerns for those aged 19–24. Within each panel, concerns are separated by gender. (DOCX) [file pone.0323199.s006.docx]

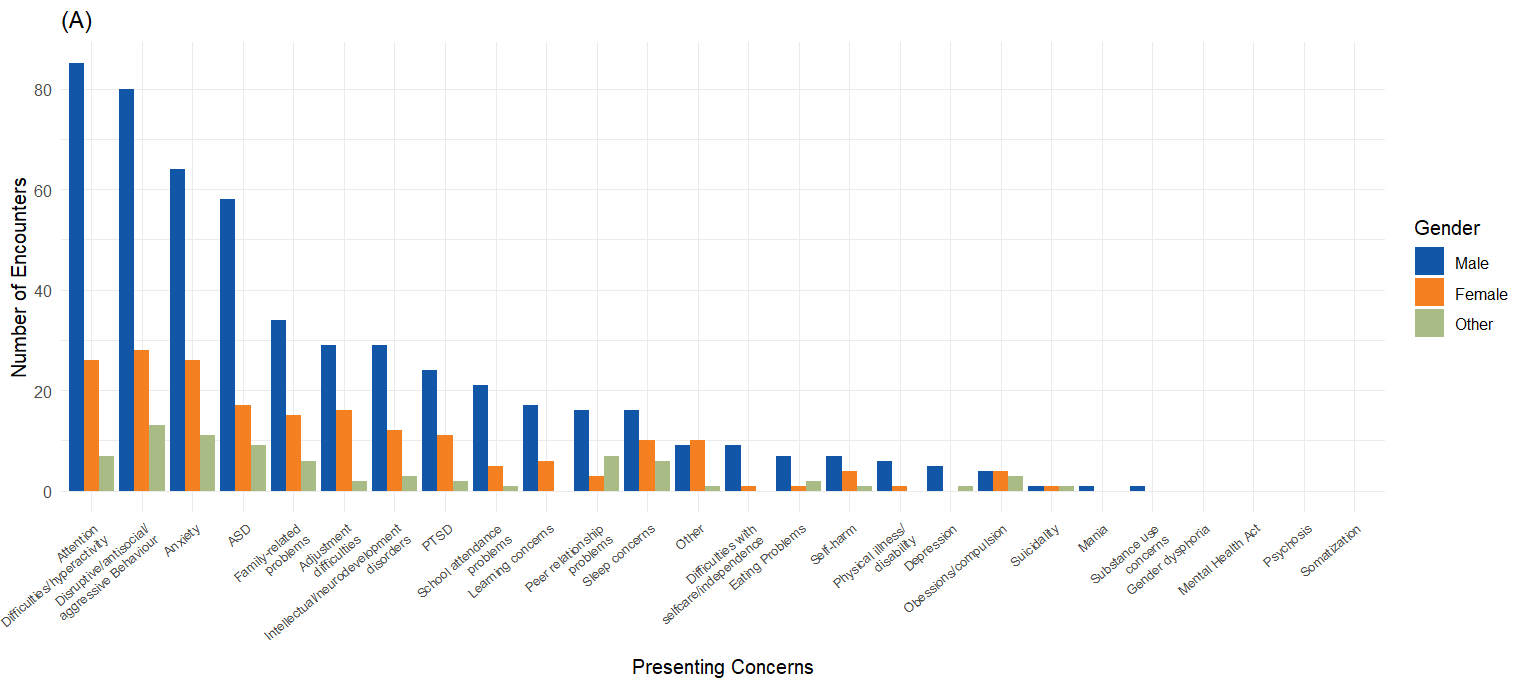

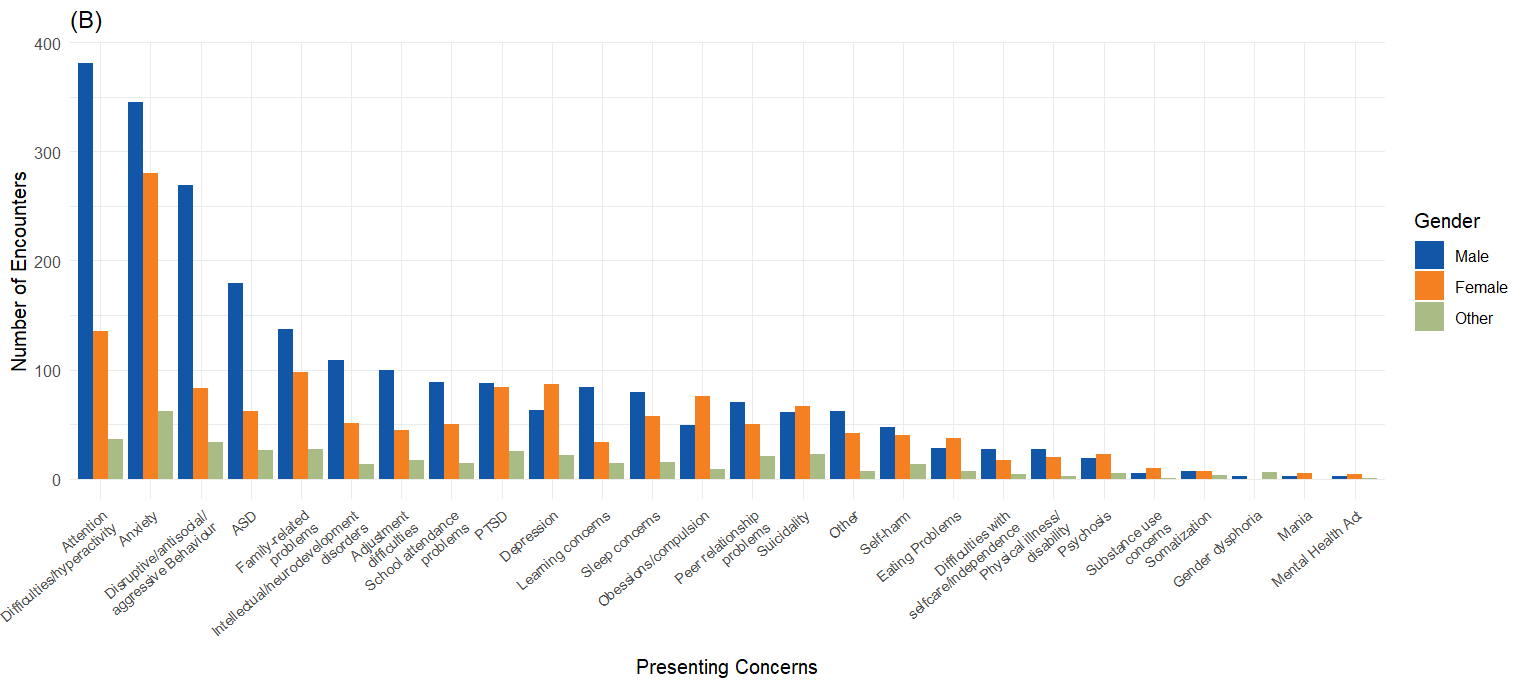

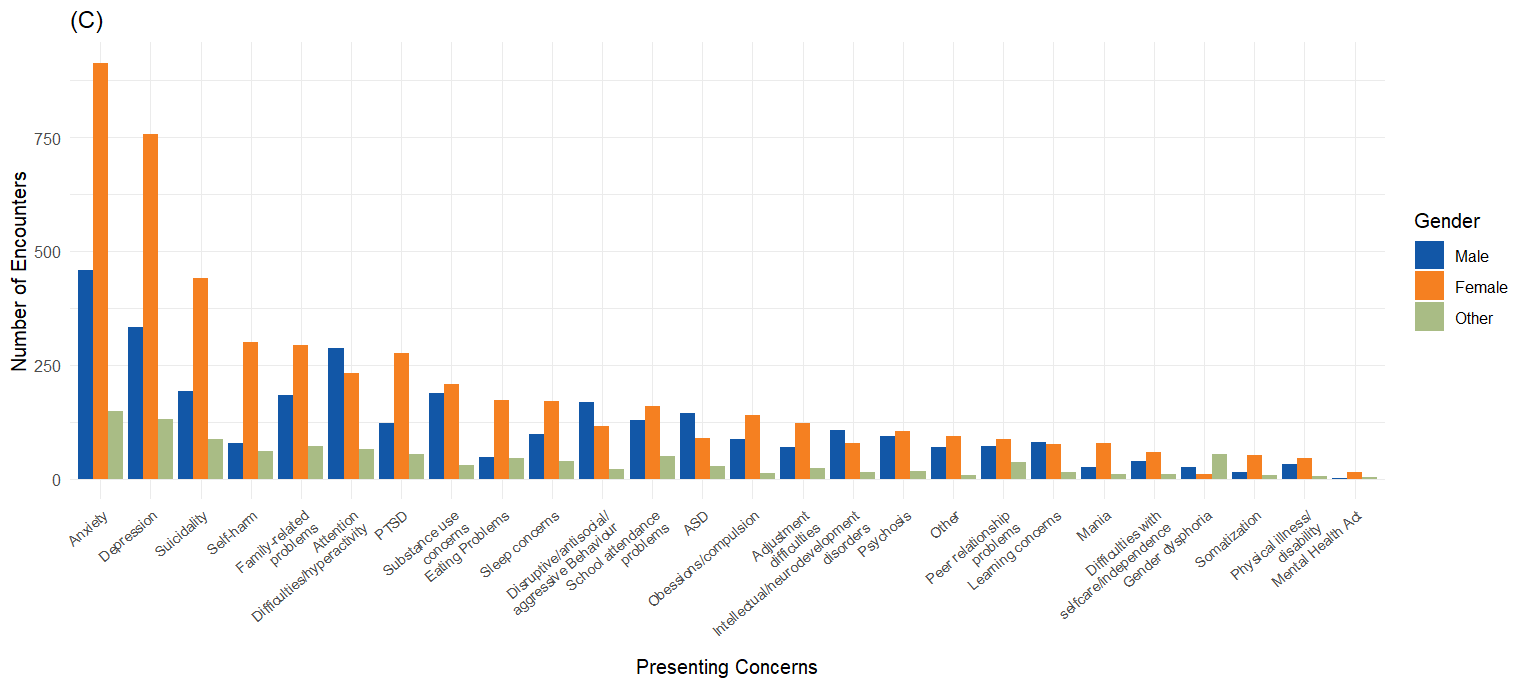

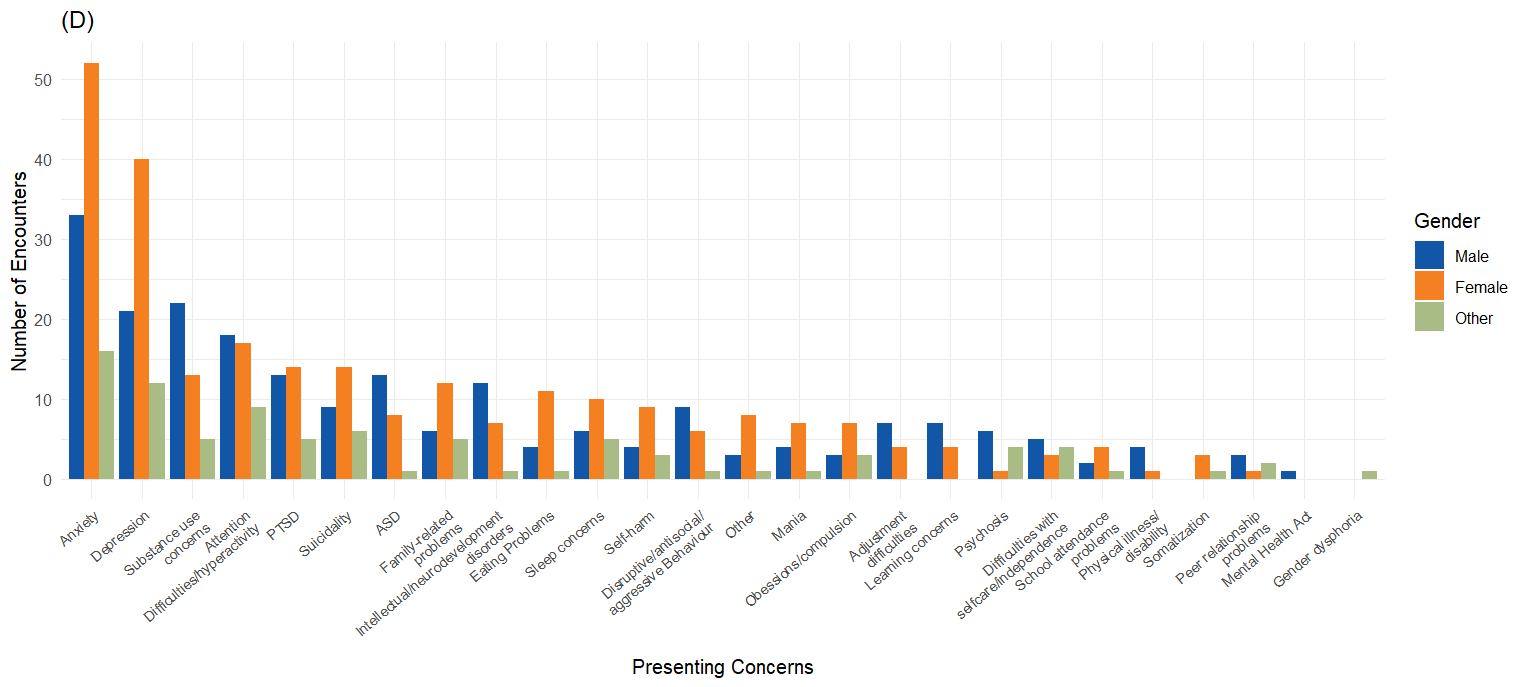


**Supplementary figure 6 (A-D). Patient concerns separated by age range and gender. Panel (A) shows the most common patient concerns for patients aged 1-6, panel (B) shows the most common patient concerns for patients aged 7-12, panel (C) shows common patient concerns from those aged 13-18, and panel (D) shows the common patient concerns for those aged 19-24. Within each panel, concerns are separated by gender.**
